# Cross-Cultural Adaptation and Validation of the Hong Kong Version of the Knee Injury and Osteoarthritis Outcome Score (HK-KOOS) for Patients with Knee Osteoarthritis

**DOI:** 10.1155/2019/8270637

**Published:** 2019-08-14

**Authors:** Andy S. K. Cheng, Ka-chun Chan, Sum-yuet Chan, Miu-kwan Fan, Man-kwan Fung, Oi-yan Lee, Cindy T. T. Kwok, Jackson K. K. Wong

**Affiliations:** ^1^Department of Rehabilitation Sciences, The Hong Kong Polytechnic University, Hung Hom, Kowloon, Hong Kong; ^2^Department of Occupational Therapy, Pok Oi Hospital, Au Tau, Yuen Long, Hong Kong; ^3^SAHK, Pak Tin Preschool Centre, Kowloon, Hong Kong; ^4^Department of Occupational Therapy, Tuen Mun Hospital, 23 Tsing Chung Koon Road, Tuen Mun, Hong Kong; ^5^Department of Occupational Therapy, Kwai Chung Hospital, 3-15 Kwai Chung Hospital Road, Kwai Chung, NT, Hong Kong; ^6^PLK Padma & Hari Harilela Integrated Rehabilitation Centre, 310 Kwai Shing Circuit, Kwai Chung, NT, Hong Kong; ^7^Department of Occupational Therapy, United Christian Hospital, 130 Hip Wo Street, Kwun Tong, Hong Kong

## Abstract

**Introduction:**

This study aimed to validate the Hong Kong version of the Knee Injury and Osteoarthritis Outcome Score (HK-KOOS) for patients with knee osteoarthritis.

**Methods:**

Content validity was assessed using the Item and Scale Content Validity Index (I-CVI and S-CVI). Test-retest reliability and internal consistency were assessed by the Intraclass Correlation Coefficient (ICC) and Cronbach's alpha. Dimensionality was assessed by performing exploratory factor analysis (EFA). Convergent and Divergent Validity was performed by examining the correlation between the HK-KOOS and the Chinese version of the Short Form 12 (SF-12) Health Survey, the Chinese Modified Barthel Index (C-MBI), and the Visual Analogue Scale for Pain (VAS-Pain). Ceiling and floor effects were also examined.

**Results:**

A total of 125 participants were recruited in this study. In general, all instructions, items, and response options were considered as understandable, indicating a satisfactory cross-cultural adaptation. The I-CVI and S-CVI scores were 0.80-1 and 0.90-1, respectively, indicating excellent content validity in terms of relevance, representativeness, and understandability. The test-retest reliability of all HK-KOOS subscales was satisfactory with ICC exceeding 0.70 for all domains. Cronbach's alpha exceeded 0.80 for all subscales, indicating satisfactory internal consistency. Medium to strong correlations were found between the HK-KOOS and the VAS-Pain, SF-12, and C-MBI. However, factor analysis indicated a seven-factor structure, rather than the original five-factor structure. Items on pain and activities of daily living were loaded in the same factors. A floor effect was present in the sports and recreation subscale.

**Discussion and Conclusions:**

Future studies should further examine the dimensionality of the KOOS. The HK-KOOS is a culturally adapted, reliable, and valid outcome measure instrument to be used in Hong Kong patients with primary knee osteoarthritis.

## 1. Introduction

Osteoarthritis (OA) is a condition characterized by focal areas of loss of articular cartilage within the synovial joints, associated with hypertrophy of the bone and thickening of the capsule [1, 2]. It is the most prevalent joint disease and a leading source of chronic pain and disability in the most developed and developing nations across the world [3, 4]. It is also a common cause of disability in older adults [5]. Knee OA is a major subgroup of articular degenerative disease and the major cause of functional limitations when performing any task involving the lower limbs [6]. In the United States, the prevalence of knee OA was 16% in the postindustrial era (late 1900s to early 2000s), which was 2.1 times higher than in the early industrial era (1800s to early 1900s) [7]. In Hong Kong, an increasing trend was observed in the total number of knee arthroplasties for primary knee OA. The number and proportion of patients older than 80 years showed an increase in knee OA from 4.8% between 2000 and 2004 to 13.8% between 2005 and 2009 [8]. Moreover, knee OA accounted for more than 80% of the disease's total impact in the United States [4].

OA is the main cause of limitations in activities; difficulties in walking, carrying objects, and dressing; and the need for human assistance [9]. Knee OA has a significant impact on multiple dimensions of people's health-related quality of life (HRQoL), as compared with healthy controls [10–13]. A reliable and valid outcome measure is important to evaluate the effectiveness of knee-specific treatments, predict treatment outcomes, and triage patients to appropriate interventions [14] particularly for the occupational therapists in Hong Kong. There are nine self-reported instruments that assess the function of the knee, as specified in Collin et al.'s review [15]. Among them, the Western Ontario and McMaster Universities Osteoarthritis Index (WOMAC) and the Knee Injury and Osteoarthritis Outcome Score (KOOS) are commonly used by both researchers and clinicians. The KOOS has been translated and validated in more than 30 languages [15]. The Singapore-Chinese version of the KOOS was culturally adapted from the English version following forward and backward word translation by two bilingual translators proficient in English and Chinese and with the input from the developer. It demonstrated acceptable psychometric properties in a multiethnic population with knee OA [16]. However, the Singapore-Chinese version of the KOOS could not be applied directly in Hong Kong. In terms of spoken language, the majority of Singaporeans speak Mandarin, while the Hong Kong people speak Cantonese [17, 18]. In terms of written language, most of Singaporeans write in simplified Chinese, while the Hong Kong people use traditional Chinese. The cultural adaptation of the Singapore-Chinese version of the KOOS into the HK-KOOS is necessary. The aim of this study is to cross-culturally adapt and validate the KOOS for patients with knee OA in Hong Kong. The objectives are as follows: (1) to cross-culturally adapt the Singapore-Chinese version of the KOOS into the HK-KOOS for patients with knee OA in Hong Kong and (2) to examine the psychometric properties, such as content validity, test-retest reliability, construct validity, and internal consistency, of the HK-KOOS.

## 2. Materials and Methods

Ethical approval was obtained from the Departmental Research Committee of the Department of Rehabilitation Sciences, Hong Kong Polytechnic University, and the Ethical Committee of the Hong Kong Hospital Authority. Informed consent was obtained from the participants prior to the study commencing. Participants with knee OA were recruited from the Department of Occupational Therapy of two public hospitals in Hong Kong from August to November 2015 by convenient sampling.

### 2.1. Inclusion and Exclusion Criteria of the Participants

The inclusion criteria were those patients who (1) had been diagnosed with knee OA by their attending orthopedic surgeon, based on clinical and radiographic features; (2) were 18 years old or above; (3) were able to understand Cantonese; and (4) were able to read and understand simple questions. The exclusion criteria were those patients who (1) were unable to give written consent; (2) suffered from other physical disabilities, which may cause functional limitations when performing any task involving the lower limbs; and (3) suffered from psychiatric problems.

A total of 125 participants were recruited to complete the questionnaire. Of those, 35 were randomly selected for test-retest reliability. Sample size was calculated based on previous similar reliable and valid KOOS studies and medium effect size indexes, which represented an average size of observed effects from calculated sample sizes [19]. Moreover, the sample size was comparable with the original Swedish-English, German, and French versions of KOOS [20–22].

### 2.2. KOOS

It is a self-administered health-related quality of life (HRQoL) outcome measure for patients with knee OA. It was developed based on the WOMAC Osteoarthritis Index Likert version 3.0 [20]. The KOOS consists of five domains with 42 items, including pain frequency and severity, symptoms, difficulty experienced during activities of daily living (ADL), sports and recreation (Sports/Rec), and knee-related quality of life (QoL) [15]. Each item is rated on a 5-point Likert scale ranging from 0 (lease severe) to 4 (most severe). A normalized score is calculated for each domain ranging from 0 to 100. A lower score indicates more extreme knee problems and poor functional status [23]. It takes around 10 minutes to complete. The KOOS demonstrated a good validity and reliability and demonstrated high levels of responsiveness in reflecting the severity of knee OA [15, 24, 25].

### 2.3. Cross-Cultural Adaptation

Cross-cultural adaptation explored cultural adaptation and language issues that arise when developing an instrument in different settings [26]. Occupational therapists and permanent residents in Hong Kong who are proficient in written traditional Chinese and spoken Cantonese were invited to adapt the Singapore-Chinese KOOS in order to make it relevant to Hong Kong culture by reviewing the instructions, items, and response options. Chinese characters, Cantonese colloquialisms, and grammar were considered during the adaptation process. Both the Singapore-Chinese and original-English KOOS were provided for reference during this process.

### 2.4. Content Validity

Content validity was used to examine if the instrument can be cross-culturally adapted for application in Hong Kong [27]. Three expert panels (*n* = 15) were formed to assess the relevance, representativeness, and understandability of the Hong Kong version. The relevance panel consisted of five occupational therapists with at least three years of working experience in orthopedics and knee rehabilitation. The representativeness panel consisted of five occupational therapists with at least one year of working experience in orthopedics and knee rehabilitation. The understandability panel was formed by five permanent residents of Hong Kong who are proficient in written traditional Chinese and spoken Cantonese.

To achieve excellent content validity, the Item-level Content Validity Index (I-CVI) should be ≥0.78 and the Scale-level Content Validity Index (S-CVI) should be ≥0.90 [28]. Items with I − CVI < 0.78 were revised based on the comments and suggestions of experts. The final version was adopted for reliability and construct validity testing. The original English version of KOOS is shown in the Appendix.

### 2.5. Test-Retest Reliability

Test-retest reliability is the degree to which a person provides similar answers to repeated measures over time [29]. The Intraclass Correlation Coefficient (ICC) (two-way mixed effects model, single measure) with a 95% confidence interval (CI) was used to assess test-retest reliability, with ICC ≥ 0.70 considered as satisfactory [30]. Moreover, the standard error of measurement (SEM) and the minimal detectable change (MDC) were used to evaluate the responses of truly unchanged patients when the instrument is conducted on two occasions and to distinguish the true performance changes regardless of the measurement error. SEM was calculated using the following formula: $\text{SEM}=\text{SD}*\sqrt{1-\text{ICC}}$. MDC was calculated using the following formula: $\text{MDC}=1.96*\sqrt{2}*\text{SEM}$ [31]. The test-retest interval was set between seven and 14 days, with reference to the Swedish and French versions of KOOS [20, 21].

### 2.6. Testing of the Psychometric Properties of the HK-KOOS

#### 2.6.1. Dimensionality

Exploratory factor analysis (EFA), using a maximum likelihood (ML) extraction method with Promax (oblique) rotation was performed to determine the factor structure of each domain. The following criteria were used to determine the factor and item reduction: (1) the Kaiser-Meyer-Olkin Test (KMO) of a factor must be greater than .05, (2) the Bartlett's Test of Sphericity result for a factor must be below .05, (3) each factor must have an eigenvalue of 1 or above, and (4) items must have a factor loading of 0.3 or above [29].

#### 2.6.2. Construct Validity

Convergent validity and divergent validity were assessed using the Pearson correlation coefficient, with ≤0.25 indicating low correlation, 0.25-0.50 showing fair to moderate correlation, 0.50-0.75 demonstrating moderate to good correlation, and ≥0.75 showing good to excellent correlation [32]. Participants were asked to complete the HK-KOOS, the Chinese version of the Short Form 12 Health Survey (SF-12), the Chinese Modified Barthel Index (C-MBI), and the Visual Analogue Scale for Pain (VAS-Pain). These measures were selected as they measure similar qualities in KOOS and based on their having satisfactory construct validity and reliability. We also chose them since they had available Chinese versions in Hong Kong.

*(1) SF-12*. The Chinese version of the SF-12 is a validated generic HRQoL instrument containing 12 items. It measures HRQoL through Physical Component Summary (PCS) and Mental Component Summary (MCS) domains, with higher scores reflecting better HRQoL [33].

*(2) C-MBI*. C-MBI is a widely used ADL assessment that has been validated in HK [34]. A total of 10 ADLs, such as bathing and feeding, are included. It is measured on a 5-point Likert scale; 1 represents unable to perform a task while 5 represents fully independent. The full score of the MBI is 100. A lower MBI score indicates more dependence.

*(3) VAS-Pain*. The VAS-Pain is a single-item scale that measures current pain intensity [35]. The total scores range from 0 to 100, with 0 indicating no pain and 100 indicating severe pain. A higher score indicates greater pain intensity [36]. Cut-off points of the VAS-Pain are recommended as follows: no pain (0-4), mild pain (5-44), moderate pain (45-74), and severe pain (75-100) [36]. This linear analogue scale was found to be suitable and applicable in the Chinese population [37].

#### 2.6.3. Internal Consistency

Internal consistency assesses the extent to which items in a scale are correlated and measure the same concept [30]. Cronbach's alpha was used to examine whether or not the items in each scale belonged to the correct hypothesized subscale. A Cronbach's alpha of 0.70-0.95 was considered to be satisfactory [30].

### 2.7. Floor and Ceiling Effect

Floor or ceiling effects appear when more than 15% of the participants reach the highest or lowest score [38], which, in this case, may affect the reliability, validity, and responsiveness of the HK-KOOS. All statistical analyses were performed using IBM SPSS statistical software version 21. The level of significance was set as *p* ≤ 0.05.

## 3. Results

The demographics of participants are shown in Table 1. The majority of the participants were female (69.6%), received primary education (65.6%), were retirees (51.2%), and had a duration of onset of knee OA between one and five years (42.4%). The means, standard deviations, and score ranges of the KOOS subscales are shown in Table 2.

### 3.1. Cross-Cultural Adaptation and Content Validity

Simplified Chinese characters were converted into traditional Chinese characters. Discussions were raised for items A16, A17, P2, SP4, and Q4, as the Mandarin and Cantonese expressions of “intensity of housework” and “knee joint” were different. The wordings were replaced with culturally relevant Cantonese translations. Three groups of expert panels (*n* = 15) were recruited for content validity testing. For relevance, I-CVI and S-CVI were 1. For representativeness, most of the I-CVI ranged from 0.80 to 1. For understandability, most of the I-CVI ranged from 0.80 to 1. These indicated excellent content validity in terms of relevance, representativeness, and understandability.

### 3.2. Test-Retest Reliability

A total of 35 participants were randomly selected to complete the questionnaire for a second time. The ICC of the five domains ranged from 0.76 to 0.86, which indicated good to excellent test-retest reliability. The MDC ranged from 3.86 to 12.06 (Table 3).

### 3.3. Dimensionality

Exploratory factor analysis (EFA) was examined for the whole sample (*n* = 125). The maximum likelihood extraction method with Promax (oblique) rotation was performed. The KMO was 0.91 and Bartlett's Test of Sphericity was significant (*χ*^2^(861) = 4470.695, *p* < .05). The EFA indicated that a seven-factor solution accounted for 70.71% of variance (Factor 1: 45.91%; Factor 2: 7.33%; Factor 3: 5.10%; Factor 4: 3.73%; Factor 5: 3.34%; Factor 6: 2.80%; and Factor 7: 2.50%). The eigenvalues were 19.28, 3.08, 2.14, 1.57, 1.40, 1.18, and 1.05, respectively. The Scree Plot also supported the seven-factor solution. Items P1 and P2 were removed due to low-factor loading (<.03) (Table 4).

### 3.4. Construct Validity

The Pearson product-moment correlation was conducted to measure the linear correlation between the KOOS subscale and the selected measures. KOOS pain, ADL, Sports/Rec, and QoL scores demonstrated fair to good correlation with the VAS-Pain, the C-MBI, the SF-12 Physical Component Summary (PCS) scale score, and the SF-12 Mental Component Summary (MCS) scale score. The KOOS symptoms only had correlations with the VAS-Pain, SF-12 PCS, and SF-12 MCS scores (*r* = −.357‐.459, *p* < 0.01). The KOOS pain score demonstrated the strongest correlation with the VAS-Pain score (*r* = −.621, *p* < 0.01), while the KOOS Sports/Rec score demonstrated the weakest correlation with the SF-12 MCS score (*r* = .209, *p* < 0.05) (Table 5).

### 3.5. Internal Consistency

Cronbach's alpha was used to assess the internal consistency of the KOOS. Two models were performed, one for the original factor structure and one for the seven-factor structure. The Cronbach's alpha of the original version of the KOOS subscales ranged from 0.80 to 0.96, which indicated good internal consistency. The Cronbach's alpha of the seven-factor structure ranged from 0.80 to 0.94 (Table 6).

### 3.6. Floor and Ceiling Effects

A ceiling effect (indicating the best possible score) was not present in the HK-KOOS, as none of the participants scored the highest score in any of the subscales. A floor effect (indicating the worst possible score) was not present in the subscales of pain, symptoms, and ADL and QoL. However, it was found in the Sports/Rec subscale, with 20% of participants scoring the lowest score in this subscale (Table 2).

## 4. Discussion

An occupational therapist, as one of the members in a multidisciplinary team, plays an important role in the management of knee OA and rehabilitation following surgery, e.g., total knee replacement (TKR). The rehabilitation goal is to promote functional recovery and facilitate safe and early discharge through reliable and valid preoperative assessments and postoperative education, functional training, provision of assistive devices, and/or home modification. The occupational therapist would conduct various functional assessments to evaluate the rehabilitation outcomes and monitor the treatment program. The health status and health outcome perceived by a patient were widely considered as an essential component in outcome evaluation. Preoperative pain and functional status, as measured by patient-reported outcome measures, have been shown to predict pain and functional ability after TKR [39].

The results of the present study indicated that the HK-KOOS was a reliable and valid measure of HRQoL for patients with knee OA. The HK-KOOS was successfully cross-culturally adapted in HK, as shown by the satisfactory results of the reliability and validity tests. For content validity, I-CVI and S-CVI ranged from 0.80-1 to 0.90-1, respectively. This revealed that the HK-KOOS had excellent content validity in the areas of relevance, representativeness, and understandability. In terms of test-retest reliability, acceptable ICC was found in all subscales. The overall results were consistent with those of other validation studies, including Singapore-Chinese (ICC = 0.60‐0.87) and Persian (ICC = 0.61‐0.91) [40]. However, the MDC values ranged from 3.86 to 12.06 for the five subscales, which is different from the previous studies [41–43]. The MDC values were varied between studies. The MDC values ranged from 2.2 to 2.9 for the Polish version, and the MDC values ranged from 2.2 to 4.4 for the Finnish version [41, 42]. Further validations are suggested to explain the inconsistency between studies.

In terms of internal consistency, Cronbach's alpha ranged from 0.80 to 0.96 for the five subscales. This result was consistent with the original Swedish (0.78-0.91), Arabic (0.80-0.96), and French versions of KOOS (0.76-0.91) [20, 21, 44] and revealed that the HK-KOOS had satisfactory internal consistency.

In terms of dimensionality, the Hong Kong version of the KOOS was loaded on seven factors, which is different from previous validation studies [41, 44–46]. Although the KOOS has been translated and validated in more than 30 languages, the factor structure is still ambiguous [47]. Up to now, there are only five studies, including the original version, that report the results of principal component analysis (PCA), exploratory factor analysis (EFA), and confirmatory factor analysis (CFA). The original Swedish and Polish versions concluded with five-factor structures [41]. The Malaysian version also reported a five-factor structure, with only 26 items included [46]. The Dutch and Arabic versions reported one-factor structures [44, 45]. In our study, P1 and P2 are shown to have low-factor loadings (P1: .286 and P2: .294). Moreover, most of the items regarding pain and activities of daily living were loaded on the same factor, especially factor 5 and factor 6. Previous findings suggest that items regarding pain and ADL subscales overlap. The original developer of the KOOS adopted the items from the WOMAC, and previous validation studies of the WOMAC have also found that the items in different subscales were loaded on the same factor, which implies that the original structure of the KOOS may have possible areas of overlap across different subscales [48, 49]. However, since our result is different from the original hypothesized structure, future studies should further examine the dimensionality of the KOOS.

Finally, in terms of floor and ceiling effects, 20% of participants scored the lowest score in the Sports/Rec subscale, which was comparable to the Dutch version [45]. This can be explained by the severity of knee OA with older age. It is possible that the elderly faced functional and physical limitations when taking part in the activities mentioned in the Sports/Rec domain. One previous study noted that questions in the Sports/Rec domain are more applicable to younger patients [45]. Further research should examine whether or not the existing questions in the Sports/Rec domain should be amended, with the inclusion of sports and recreational activities in which the elderly participate.

This study has a major limitation since the average age of the participants is 67.37 years old, which may make the study results not generalizable to young patients with knee OA and other knee problems. Previous studies have proven that the KOOS can be applicable to young people and patients with different knee problems, such as Anterior Cruciate Ligament and those who have undergone total knee replacement. Further validation studies of the HK-KOOS, administered to younger populations, are recommended.

Developing the HK-KOOS is of great clinical significance. First, the HK-KOOS can help occupational therapists to measure multiple dimensions of HRQoL, other than the functional outcomes of patients with knee OA. Occupational therapists could use the KOOS to conduct early screening on patients prior to performing any kind of knee treatment and intervention.

## 5. Conclusion

The HK-KOOS is a validated and reliable outcome measure for patients with knee OA. The HK-KOOS could be used as a self-reported, disease-specific instrument for those with primary knee OA in Hong Kong to evaluate both short-term and long-term consequences of knee OA. It can help occupational therapists to quantify knee-related disabilities and provide useful directions for future interventions.

## Figures and Tables

**Table 1:** Characteristics of the participants.

	*N* (%)	
	Total (*n* = 125)	Test-retest reliability participants (*n* = 35)
Age (years)		
Mean (SD)	67.37 (8.29)	65.40 (8.99)
Range	47-85	50-80
Sex		
Male	38 (30.4)	7 (20.0)
Female	87 (69.6)	28 (80.0)
Education		
No education	7 (5.6)	2 (5.7)
Primary education	82 (65.6)	24 (68.6)
Secondary education	33 (26.4)	7 (20.0)
Tertiary education	3 (2.4)	2 (5.7)
Occupation		
Retirees	64 (51.2)	14 (40.0)
Homemakers	30 (24.0)	13 (37.1)
Workers	31 (24.8)	8 (22.9)
Onset of knee OA (years)		
1-5	53 (42.4)	20 (57.1)
6-10	37 (29.6)	8 (22.9)
11-15	15 (12.0)	2 (5.7)
>15	20 (16.0)	5 (14.3)

**Table 2:** Means, standard deviations, score ranges, and the number of floor and ceiling effects of the HK-KOOS subscales (*n* = 125).

	Mean	SD	Range	Floor effect (*n*, %)	Ceiling effect (*n*, %)
KOOS pain	48.42	19.90	0-97	1 (0.8)	0 (0)
KOOS symptoms	48.98	21.26	0-100	1 (0.8)	2 (1.6)
KOOS ADL	54.19	20.67	0-94	1 (0.8)	0 (0)
KOOS sport/recreation	22.44	20.33	0-95	24 (19.2)	0 (0)
KOOS QoL	36.45	21.80	0-100	7 (5.6)	1 (0.8)

**Table 3:** Reliability indices of the HK-KOOS subscales (*n* = 35).

KOOS subscale	The 1st attempt	The 2nd attempt	Difference	ICC^a^ (95% CI)	SEM^b^	MDC_95_^c^
	Mean (SD)	Mean (SD)	Mean (SD)			
Pain	17.69 (6.11)	16.97 (6.25)	-0.71 (4.32)	0.86 (0.72, 0.93)	2.34	6.48
Symptoms	14.00 (5.25)	12.89 (4.90)	-1.11 (3.45)	0.86 (0.72, 0.93)	1.83	5.09
ADL	27.00 (11.85)	25.89 (12.56)	-1.11 (8.31)	0.88 (0.77, 0.94)	4.34	12.06
Sport/recreation	13.94 (4.15)	13.43 (4.20)	-0.51 (3.63)	0.78 (0.55, 0.89)	3.33	6.45
QoL	9.49 (2.84)	9.26 (2.84)	-0.23 (2.46)	0.76 (0.52, 0.88)	1.39	3.86

^a^Intraclass correlation coefficient. ^b^Standard error measurement. ^c^Minimal detectable change with 95% confidence.

**Table 4:** Factor loadings of each item for HK-KOOS (*n* = 125).

HK-KOOS items	1	2	3	4	5	6	7
A9	1.134						
A11	1.102						
A12	0.688						
A14	0.619						
A10	0.609						
A15	0.488						
SP4	0.413						
S7		0.758					
S6		0.758					
S3		0.691					
S1		0.552					
S2		0.49					
Q3		0.447					
SP3			0.923				
SP2			0.888				
SP5			0.694				
SP1			0.674				
A5			0.349				
A16				0.707			
A17				0.539			
Q4				0.518			
S5				0.442			
A8				0.431			
Q2				0.31			
P9					0.837		
A4					0.811		
P5					0.609		
A6					0.52		
A1						0.878	
A2						0.741	
P6						0.723	
P4						0.464	
A7						0.419	
P3							0.651
S4							0.569
A3							0.502
P8							0.429
P7							0.385
A13							0.359
Q1							0.345

Items with factor loadings under .03 were suppressed (items P1 and P2). P=pain, S=symptoms, A=activities of daily living, SP=sport and recreation, and Q=knee-related quality of life.

**Table 5:** Convergent and divergent validity of select scales (*n* = 125).

	VAS pain	MBI	SF-12 PCS	SF-12 MCS
KOOS pain	−.621^∗∗^	.329^∗∗^	.522^∗∗^	.436^∗∗^
KOOS symptoms	−.357^∗∗^	.095	.459^∗∗^	.361^∗∗^
KOOS ADL	−.535^∗∗^	.358^∗∗^	.594^∗∗^	.519^∗∗^
KOOS sport/recreation	−.430^∗∗^	.328^∗∗^	.526^∗∗^	.209^∗^
KOOS QOL	−.448^∗∗^	.376^∗∗^	.623^∗∗^	.573^∗∗^

^∗^Significant correlation at the 0.05 level (two tailed). ^∗∗^Significant correlation at the 0.01 level (two tailed).

**Table 6:** Comparison of internal consistency of the original KOOS and HK-KOOS (*n* = 125).

Original KOOS	Cronbach's alpha	HK-KOOS	Cronbach's alpha
Symptoms	0.82	Factor 1 (A9-A12, A14-A15, SP4)	0.93
Pain	0.92	Factor 2 (S1-S3, S6-S7, Q3)	0.84
ADL	0.96	Factor 3 (SP1-SP3, SP5, A5)	0.88
Sport and recreation	0.85	Factor 4 (A8, A16-A17, Q2, Q4, S5)	0.80
QoL	0.80	Factor 5 (P5, P9, A4, A6)	0.90
		Factor 6 (A1-A2, A7, P6, P4)	0.91
		Factor 7 (P3, P7-P8, A3, A13, S4, Q1)	0.86

## Data Availability

The data used to support the findings of this study are available from the corresponding author upon request.

## Appendix

### Original English Version of KOOS

*Instructions*:

This survey asks for your view about your knee. This information will help us keep track of how you feel about your knee and how well you are able to do your usual activities. Answer every question by ticking the appropriate box, only one box for each question. If you are unsure about how to answer a question, please give the best answer you can.

*Symptoms*

These questions should be answered thinking of your knee symptoms during the *last week*.

S1. Do you have swelling in your knee?

□ Never □ Rarely □ Sometimes □ Often □ Always

S2. Do you feel grinding/friction, hear clicking/cracking, or any other type of noise when your knee moves?

□ Never □ Rarely □ Sometimes □ Often □ Always

S3. Does your knee jam or lock when moving?

□ Never □ Rarely □ Sometimes □ Often □ Always

S4. Can you straighten your knee fully?

□ Always □ Often □ Sometimes □ Rarely □ Never

S5. Can you bend your knee fully?

□ Always □ Often □ Sometimes □ Rarely □ Never

*Stiffness*

The following questions concern the amount of joint stiffness you have experienced during the *last week* in your knee. Stiffness is a sensation of restriction or slowness in the ease with which you move your knee joint.

S6. How severe is your knee joint stiffness after first wakening in the morning?

□ None □ Mild □ Moderate □ Severe □ Extreme

S7. How severe is your knee stiffness after sitting, lying, or resting *later in the day*?

□ None □ Mild □ Moderate □ Severe □ Extreme

*Pain*

P1. How often do you experience knee pain?

□ Never □ Monthly □ Weekly □ Daily □ Always

What amount of knee pain have you experienced in the *last week* during the following activities?

P2. Twisting/pivoting on your knee

□ None □ Mild □ Moderate □ Severe □ Extreme

P3. Straightening knee fully

□ None □ Mild □ Moderate □ Severe □ Extreme

P4. Bending knee fully

□ None □ Mild □ Moderate □ Severe □ Extreme

P5. Walking on flat surface

□ None □ Mild □ Moderate □ Severe □ Extreme

P6. Going up or down stairs

□ None □ Mild □ Moderate □ Severe □ Extreme

P7. At night while in bed

□ None □ Mild □ Moderate □ Severe □ Extreme

P8. Sitting or lying

□ None □ Mild □ Moderate □ Severe □ Extreme

P9. Standing upright

□ None □ Mild □ Moderate □ Severe □ Extreme

*Function, daily living*

The following questions concern your physical function. By this, we mean your ability to move around and to look after yourself. For each of the following activities, please indicate the degree of difficulty you have experienced in the *last week* due to your knee.

A1. Descending stairs

□ None □ Mild □ Moderate □ Severe □ Extreme

A2. Ascending stairs

□ None □ Mild □ Moderate □ Severe □ Extreme

A3. Rising from sitting

□ None □ Mild □ Moderate □ Severe □ Extreme

A4. Standing

□ None □ Mild □ Moderate □ Severe □ Extreme

A5. Bending to floor/pick up an object

□ None □ Mild □ Moderate □ Severe □ Extreme

A6. Walking on flat surface

□ None □ Mild □ Moderate □ Severe □ Extreme

A7. Getting in/out of car

□ None □ Mild □ Moderate □ Severe □ Extreme

A8. Going shopping

□ None □ Mild □ Moderate □ Severe □ Extreme

A9. Putting on socks/stockings

□ None □ Mild □ Moderate □ Severe □ Extreme

A10. Rising from bed

□ None □ Mild □ Moderate □ Severe □ Extreme

A11. Taking off socks/stockings

□ None □ Mild □ Moderate □ Severe □ Extreme

A12. Lying in bed (turning over, maintaining knee position)

□ None □ Mild □ Moderate □ Severe □ Extreme

A13. Getting in/out of bath

□ None □ Mild □ Moderate □ Severe □ Extreme

A14. Sitting

□ None □ Mild □ Moderate □ Severe □ Extreme

A15. Getting on/off toilet

□ None □ Mild □ Moderate □ Severe □ Extreme

A16. Heavy domestic duties (moving heavy boxes, scrubbing floors, etc.)

□ None □ Mild □ Moderate □ Severe □ Extreme

A17. Light domestic duties (cooking, dusting, etc.)

□ None □ Mild □ Moderate □ Severe □ Extreme

*Function, sports and recreational activities*

The following questions concern your physical function when being active on a higher level. The questions should be answered thinking of what degree of difficulty you have experienced during the *last week* due to your knee.

SP1. Squatting

□ None □ Mild □ Moderate □ Severe □ Extreme

SP2. Running

□ None □ Mild □ Moderate □ Severe □ Extreme

SP3. Jumping

□ None □ Mild □ Moderate □ Severe □ Extreme

SP4. Twisting/pivoting on your injured knee

□ None □ Mild □ Moderate □ Severe □ Extreme

SP5. Kneeling

□ None □ Mild □ Moderate □ Severe □ Extreme

*Quality of Life*

Q1. How often are you aware of your knee problem?

□ Never □ Monthly □ Weekly □ Daily □ Constantly

Q2. Have you modified your lifestyle to avoid potentially damaging activities to your knee?

□ Not at all □ Mildly □ Moderately □ Severely □ Extremely

Q3. How much are you troubled with lack of confidence in your knee?

□ Not at all □ Mildly □ Moderately □ Severely □ Extremely

Q4. In general, how much difficulty do you have with your knee?

□ None □ Mild □ Moderate □ Severe □ Extreme
